# Reframing involuntary treatment as a sentinel event: a model for improving rights-based mental health care

**DOI:** 10.3389/fpsyt.2025.1712756

**Published:** 2026-01-07

**Authors:** Giulia Cossu, Michela Atzeni, Thurayya Zreik, Massimo Tusconi, Mauro Giovanni Carta

**Affiliations:** 1 Department of Medical Sciences and Public Health, University of Cagliari, Monserrato Blocco I Cagliari (CA), Cagliari, Italy; 2 Center of Liaison Psychiatry and Psychosomatics, University Hospital of Cagliari, Cagliari, Italy; 3 Justice for Mental Health Service User Association, Beirut, Lebanon; 4 PhD Program in Capacities Building for Global Health, University of Cagliari, Cagliari, Italy; 5 PhD Program in Tropical Medicine, Universidad Popular del Cesar, Valledupar, Colombia; 6 Department of Nursing, Universidad Popular del Cesar, Valledupar, Colombia

**Keywords:** human rights, mental health, sentinel event, quality of care, coercive measures

## Abstract

**Background:**

The UN Convention on the Rights of Persons with Disabilities (CRPD) has sparked debates on psychosocial disabilities, particularly Article 12, which guarantees legal capacity without restrictions. The CRPD Committee opposes involuntary treatment and strongly advocates for support mechanisms to ensure autonomy. This raises questions about decision-making in psychiatric care and the role of involuntary treatment. Advocacy groups, which push for the elimination of involuntary treatment in favor of alternative measures, argue that involuntary treatment results from inadequate resources, while psychiatric associations highlight ethical concerns about withholding care and emphasize risks to service users and others.

**Methods:**

This article presents a model that reframes involuntary treatment as a preventable sentinel event. The approach outlines the components of a monitoring and quality improvement system, including structured reporting, root cause analysis, and co-designed interventions involving service staff members and service users.

**Results:**

The application of this model may identify key structural and systemic drivers of involuntary treatment, such as insufficient community-based services, lack of training, and power asymmetries. It also highlights the potential of participatory governance mechanisms and user-led monitoring to foster accountability and drive rights-based reforms.

**Conclusion:**

This approach may help align mental health services with CRPD principles, reduce involuntary treatment, and enhance accountability, legitimacy, and foster collaborative relationships between users and providers. It may also help overcome the current impasse around coercive practices by enabling the identification and analysis of the structural and cultural mechanisms that sustain them over time, thereby opening up new possibilities for their management and overcoming.

## 1 Introduction

The United Nations Convention on the Rights of Persons with Disabilities (CRPD) (1), has given rise to heated debates in the field of psychosocial disabilities (2, 3). Among the most prominent of these debates is the discussion surrounding the interpretation of Article 12 of the Convention, and specifically, the General Comment on Article 12, which stipulates that the legal capacity of persons with disabilities cannot be denied or restricted under any circumstances (4). According to Article 12 of the CRPD, “States shall take appropriate measures to provide access by persons with disabilities to the support they may need in exercising their legal capacity” (1).

The Committee on the Rights of Persons with Disabilities is “the body of independent experts which monitors implementation of the Convention by the States Parties” (5). This body interprets Article 12 of the Convention as a preclusion to any involuntary intervention. (6). The committee’s activities impose specific legal constraints on nations that have signed the treaty: signatory states are required to align their national policies with the treaty, and those that have also signed the additional protocols are obligated to undergo monitoring processes.

Coercive measures, in mental health hospital as well as in the community (7), including involuntary treatment, can be defined as any practice that “involves the use of authority to restrain another’s autonomy” (8). Some authors referring to such measures as a “continuum” with “gray areas” that include practices ranging from indirect suggestion and pressure to involuntary hospitalization, seclusion, and restraint (9). In the literature, the terminology used to describe coercive practices is not uniform, and different terms such as “involuntary treatment”, “compulsory treatment”, and broader expressions like “coercive measures” are sometimes used interchangeably, although they do not always denote identical concepts (10). For the purposes of this manuscript, we use the term “involuntary treatment” as the overarching concept referring to interventions delivered without the person’s free and informed consent. We retain the term “compulsory treatment” only when citing legal models or studies that explicitly use this wording. More broadly, we refer to “coercive practices” when discussing the wider continuum of actions that may restrict autonomy, as described in the literature (8, 9). Clarifying this terminology is essential to avoid conceptual ambiguity and to ensure consistency throughout the manuscript (10).

The CRPD’s respect and recognition for legal capacity and the CRPD Committee’s subsequent position on the elimination of coercive measures such as involuntary treatment in mental health care has prompted different interpretations and further ambiguity. Despite the extensive debate inspired by the CRPD and its General Comment No. 1, several key questions remain unresolved for a wide range of stakeholders, including policymakers, clinicians, legal and human rights bodies, and service user advocacy groups. These actors often hold differing interpretations of the CRPD’s implications, particularly with regards to decision-making, risk assessment, and the permissibility of involuntary interventions. As a result, the following questions continue to drive disagreement and remain insufficiently answered:

1. How far can the “support” for exercising legal capacity go?
2. To what extent can a person with a mental health condition, who is deemed (by themselves or others) a threat to themselves or others, refuse treatment? In other words, does the right to health justify limiting an individual’s autonomy over their own body and health decisions?
3. Who has the authority to decide what is best for a person? Doctors come from a long tradition in which it was permissible to decide on behalf of the service user, for example some decades ago, it was even acceptable to hide a diagnosis when the doctor felt that this was the best interest of the user.
4. On what scientific basis (with what predictive accuracy) can one judge the social dangerousness induced by a psychopathological condition in a person?
5. How “protective” is an involuntary treatment or admission for a user’s health when it has been consistently demonstrated that involuntary treatment can act as a significant negative determinant in the trust in the care system, which, in turn, could adversely impact the user’s voluntary adherence to and awareness of future treatments, and potentially undermine the very goals of therapeutic intervention?

This debate has underscored a clear divide between various psychiatric associations and some mental health user groups and associations for civil rights, most of which have strongly advocated for a ban on coercive measures such as involuntary treatment and a shift towards more rights- and recovery-based, person-centered approaches (6, 11–13).

This position is not simply a matter of principle as the adverse consequences of involuntary treatment have been consistently recognized and highlighted in the literature. In fact, many argue that deeming involuntary treatment as acceptable runs the risk of justifying its indiscriminate administration in all cases, including in situations where such measures could have been avoided (11–13). This perspective goes on to argue that describing types of “acceptable” involuntary treatment or admissions in certain circumstances could justify and reinforce the routine use of such involuntary measures without exploring viable alternatives. Human rights associations and users’ associations contend that a significant number of involuntary health treatments and admissions could be avoided if professionals dedicated more attention and time spent to understanding the perspectives of service users and persons with psychosocial disabilities (11–13). A professional shift towards alternative non-coercive treatments, community inclusion, recovery-oriented services and in general, rights-compliant mental health services, could substantially reduce cases of involuntary treatment and admission (14–16).

This growing advocacy for rights-compliant, non-coercive approaches is further supported by a robust body of evidence highlighting the detrimental effects of involuntary treatment and admission. Research highlights the negative impacts of involuntary treatment practices on critical clinical outcomes, including on treatment compliance and service engagement (17, 18), access to services (19, 20), and hospitalization rates (21, 22). Moreover, studies indicate that there is no evidence that involuntary treatment reduces user mortality (23, 24). Furthermore, literature points to the ethical distress and potential harm experienced by clinicians when required to administer involuntary treatment (25). Despite these findings, involuntary treatment remains a common practice (26, 27). Its justification, which is frequently, though not universally, supported at institutional levels, is rooted in the belief that it protects individuals’ health and saves lives (28).

At the same time, many psychiatric advance articulate a contrasting perspective. This position is consistent with the Appelbaum’s cynical assertion that the CRPD interdicts any involuntary intervention directed at people with psychosocial disabilities, a restriction that may jeopardize the well-being of both individuals with psychosocial disabilities and those around them: “A person with dementia, unable to take care of their own needs, but unwilling to accept financial and health management, will not be forced to do so. People with depression who intend to end their life cannot be hospitalized against will … a person in the manic phase would be free to squander their savings or ruin their business. In the name of protection from discrimination, they would be free to destroy their own lives and ruin the lives of their loved ones” (6). Similarly, Freeman et al. (29) express concerns about the General Comment on Article 12 of the CRPD, arguing that it undermines the enjoyment of other rights, including the right to the highest attainable standard of health, access to justice, the right to liberty, and the right to life, for the individual, their families, and the wider population.

## 2 Are the two positions truly irreconcilable?

In order to address this divide, it is necessary to first understand the root causes of involuntary treatment, beginning with a critical exploration of the question: Why does involuntary treatment occur? The justification for involuntary treatment is often framed at the level of the service user: the decision to subject users to involuntary interventions is based on their symptoms, their perceived dangerousness, and their capacity, as assessed by professionals (30). However, this arguably narrow perspective almost entirely overlooks the environment in which these involuntary practices occur. A socio-ecological reading of coercion expands the focus to include the multiple influencing factors that play a role in forming the “ecosystem” of involuntary treatment, which most often extends far beyond the individual. Involuntary treatment does not occur in a vacuum that consists of only a user (or their symptoms) and a professional; rather, it emerges from a complex interplay of attitudinal, social, and structural barriers and facilitators. This perspective also aligns with recent developments in the field of complex systems evaluation (31–33). Lukersmith et al. proposed an ecosystem approach to assess heterogeneous mental health interventions across seven countries, emphasizing the importance of analyzing interactions between micro, meso, and macro-level determinants to evaluate system functioning and outcomes (34). Such approaches reinforce the need to look beyond individual pathology or professional decision-making and consider broader contextual and systemic factors. Applying this lens to the issue of involuntary treatment may provide a more nuanced understanding of its drivers, as well as more effective points of intervention for prevention. Exploring, identifying, and addressing these factors is key in designing a health system within which the rights of service users are respected and protected to the highest standard possible.

For example, a growing evidence base suggests that structural barriers play a key role in the prevalence of coercive practices. Research has illustrated that deficiencies in health service structures and resourcing tend to be a significant factor in the application of involuntary treatments (31). Critically considering the link between available resources and involuntary health treatments could constitute a significant step towards bridging the gap between the two positions above (31). In California, the California’s Mental Health Services Act (MHSA) (32, 35), as of fiscal year 2008-2009, distributed a large amount of tax revenues to mental health services with the aim to provide support to the people with severe mental health conditions who do not have access to adequate care. As a consequence of these measures, there was a decrease of involuntary 14-day treatments, approximately 10% below expected and a general reduction of medical visit for psychiatric emergencies (32, 35). Another example comes from Pennsylvania, where the employing resources in a combination of initiatives including response teams, staff training, data transparency, treatment malls, leadership, and advocacy, led to a significant reduction in the use of mechanical restraint and seclusion (36). These examples seem to suggest that with the allocation of adequate resources, involuntary treatments could be significantly minimized. In fact, one might speculate that in an ideal situation of unlimited resources, the use of involuntary treatments would tend to zero.

We turn once again to the CRPD as a “jumping off” point in addressing this issue: Article 4 of the CRPD explicitly acknowledges the tangible role that macro-level resources play in determining mental health outcomes for individuals. It commits signatory states to undertake measures, to the maximum of their available resources, to achieve the full realization of the rights outlined in the Convention (1). This principle does not imply that a country must commit *all* “ideal” or unlimited resources, including those it does not possess, as this could potentially undermine the fulfillment of other essential rights and needs of their citizens. Instead, the principle is that each country must make every possible effort to use all the resources at its disposal to satisfy these obligations as a matter of priority, even when the resources are scarce. The CRPD thus provides a model for understanding how systemic factors, particularly resource allocation, can influence mental health care on the individual level. The focus of our discussion, however, is on involuntary treatments which, as demonstrated, hold the potential to severely violate an individual’s human rights.

## 3 Involuntary treatment as a sentinel event

This growing body of evidence underscoring the harms of involuntary treatment, alongside ongoing debates and calls for reform, underscores the urgent need to reframe how involuntary treatment is understood and addressed within mental health systems. Taking up the call from The Lancet Psychiatry editorial titled “Whose choice is it?” (37), the authors believe there can be no alternative but to consider involuntary treatment as an error to be analyzed, or a sentinel event.

A sentinel event is an unexpected occurrence within healthcare involving death, or serious physical or psychological harm (38). Other definitions refer to unexpected or infrequent events that reveal a failure in the functioning of a service, regardless of whether significant injury occurs (31). The term sentinel refers to a systematic monitoring that may prevent similar events in the future. The US National Quality Forum defined the term serious reportable events as “preventable, serious, and unambiguous adverse events that should never occur” (never events) (39).

Within our model, we consider involuntary treatment as a sentinel event to be examined in all its forms as it occurs in the context of psychiatric inpatient care, including involuntary admissions and compulsory hospitalization procedures (such as the Italian involuntary treatment). Although these events are often legally justified, they may still have profound psychological consequences for the person and can generate moral harm by restricting autonomy and potentially undermining fundamental human rights (38). For this reason, we characterize involuntary treatment as a sentinel event due to the fact that, at least on a psychological level, it can demonstrably traumatize the person and cause even more serious moral damage in denying their human rights (40).

In this manuscript, the proposed model is intended to apply to all forms of involuntary treatment, including involuntary admissions within inpatient settings. The assumption stands that ideally, such events should not occur if appropriate and adequate resources are available. Given the seriousness of the consequences of involuntary treatment, it is necessary to monitor the root causes of the event, if all the opportunities have been evaluated and exhausted and that all the resources have been put in place so that the event could have been avoided.

## 4 Basic suggestion for a monitoring proposal

A foundational element of this model is the substantive and structured involvement of service users in the essential phases of the clinical and organizational process. Their participation is essential for ensuring that monitoring, root cause analysis, and action planning accurately reflect lived experiences and support rights-based approaches to reducing coercive involuntary treatment practices. The integration of service user involvement is described in more detail later on in this article.

**Figure 1:**
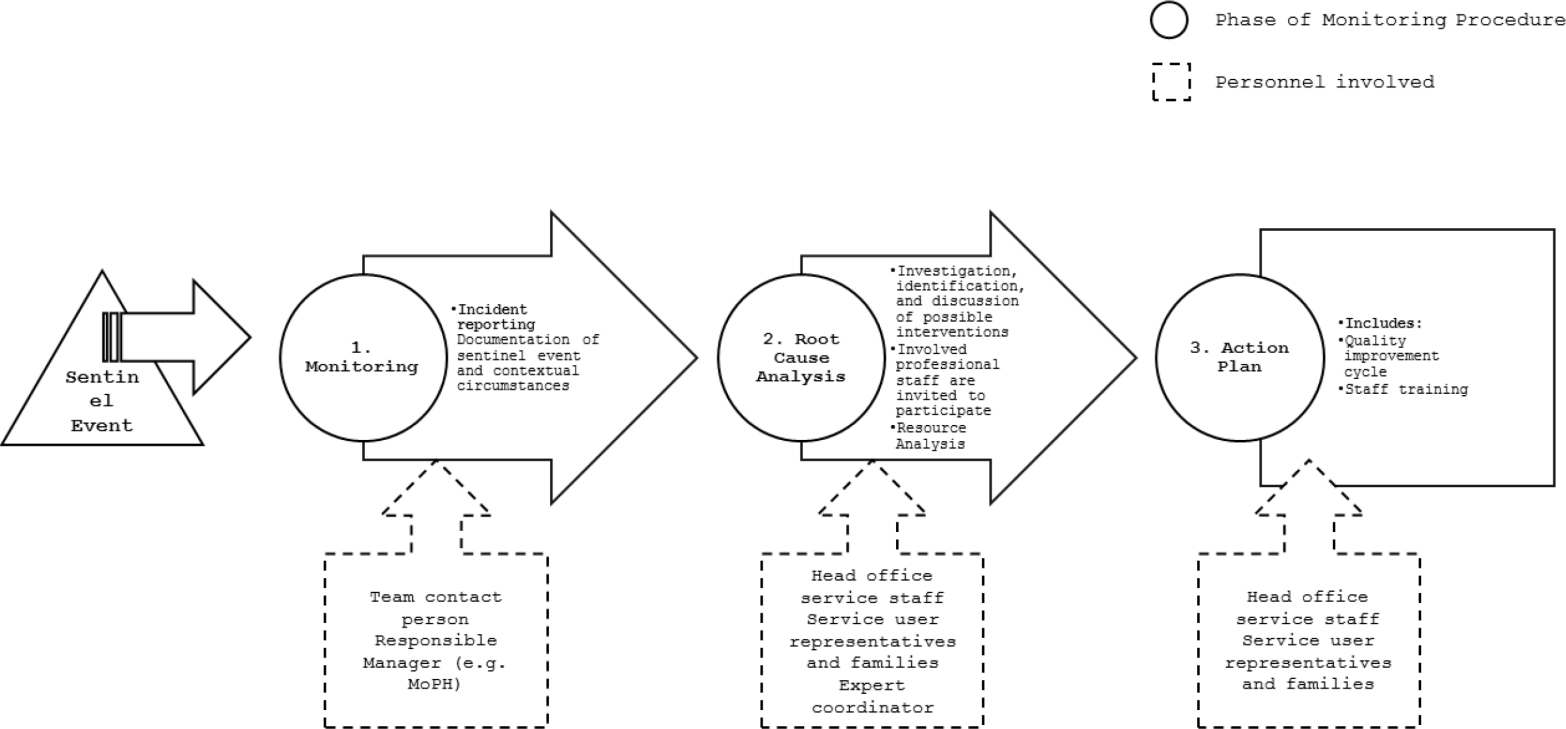
Structured monitoring process for involuntary treatment as a sentinel event.

Detecting and addressing any possible involuntary treatment as a sentinel event, with a view to improving quality, should follow a structured process. The key steps and procedures, summarized in **Figure 1**, include:

1. Monitoring;
2. Root cause analysis;
3. Action plan.

### 4.1 Monitoring

The first phase of the monitoring is meant for gathering basic details such as when the involuntary treatment occurs, the reporting of the sentinel event and the preparation of documentation for future discussion. The acquisition of data relating to sentinel event takes place by completing the “Incident Report”.

The “Incident Report” form must document:

1. Clinical causes and contextual circumstances that led to the decision to initiate involuntary treatment. Here, we note that legal provisions differ significantly across jurisdictions (26, 28, 41, 42). In several countries, including many European jurisdictions, the law provides that involuntary treatment must be implemented when the person is considered a danger to themselves or others. In other jurisdictions, such as Italy, involuntary treatment may be applied when the person is unable to provide informed consent for a treatment deemed clinically appropriate (43). In all cases, it should be specified for what reasons the treatment should not delayed and for what reasons it is essential that the treatment is carried out in the hospital if hospitalization is foreseen. Where it is legally possible to carry out the treatment in a non-hospital setting, then the specific location and reasoning must be specified.
2. Other contextual circumstances must also be described. For example: at an early stage, the staff must define whether the presence of alternative resources would have allowed the intervention to be postponed or avoided, while specifying the types of resources needed.
3. Define whether there were elements that could have avoided the involuntary treatment (e.g. presence of advanced directives or a person of support).

Importantly, hospital staff should have had sufficient capacity-building in parallel to the establishment of a monitoring system in order to be able to identify and adequately describe these causal elements and factors. In each team a contact person should be identified to manage the clinical risk, who must provide a report that must also be sent to the responsible manager (for example, the national health authority should establish a designated contact office, such as in the case of the Italian health authority), within a set period (for example 5 days from the event). However, the entire documentation (initial form filled in by the person who carried out the intervention and administrative form) should be available on site for subsequent information.

A designated contact person (or office) plays a central role in the monitoring component of the model. Drawing inspiration from models such as the Mental Health Advocate in Victoria, Australia, this role can function as an independent liaison between service users and mental health services, ensuring that reports of involuntary treatment or coercive practices are promptly registered, reviewed and followed up. Duties of such a contact point might include: receiving notifications of sentinel-events, maintaining a confidential register of such events, providing service-user support and feedback, and reporting system-wide trends to governance bodies. The contact person should ideally have sufficient independence from direct clinical management to enable impartial oversight, yet be integrated within the service governance structure to influence actions and improvements. In jurisdictions with existing advocacy mechanisms, alignment with or expansion of those roles may enhance feasibility and responsiveness (44).

### 4.2 Root cause analysis, the in-depth analysis of the sentinel event

Root cause analysis (RCA) is a structured investigation that aims to identify the causes of a problem and the actions due to eliminate it (45). It must be conducted at the head offices of the service that conducted the involuntary treatment. The protocol should include the involvement of professionals in the team, users, and family representatives, as required by the CRPD, which outlines the need for them to be consistently included in decision-making processes, particularly those that directly affect them (5), an expert coordinator and/or the designated contact person. The goal of this methodology is to establish and define:

1. Discussion of the basic causes and the exact circumstances that contributed to the event. Ideally, staff would be trained enough to be able to identify factors such as attitudinal influences, prejudgment, or even stigma as causal factors, if they were to be present.
2. Reasonable identification: the investigation must reconstruct the picture of the situation in which the event was generated and define the presence/absence of specific resources that could have helped avoid the event.
3. The enquiry must highlight the possible interventions by the team, “allied” support networks, for example social care services, service user organizations, and law enforcement agencies etc.

It is a good practice that those who have decided on and put into practice the involuntary health treatment not be present in the investigation team in the first phase of the root analysis (46, 47). In the first meeting, the flow chart of events is developed, as detailed and complete as possible. Then the next step developed in the team is also extended to those who were involved in the event. In the subsequent meetings, the aim should be to identify and envisage possible causes, contributing factors and hypothesized solutions, taking into account, when possible, the multiple layers of influences that make up the “ecosystem” of the coercive event. All this should be carried out through a structured group communication technique. In addition to analyzing qualitative interviews with key informants and reviewing relevant documentation (48), several established techniques can be applied when conducting a Root Cause Analysis (49). Crucially, members of the RCA team must be adequately trained in the specific methods and procedures required for effective RCA implementation (50).

Each element must be classified according to the degree of causality towards the involuntary treatment as:

- Not influential
- Determinant or
- Facilitator

### 4.3 Action plan

The third phase, the action plan, includes a series of strategies to make changes and reduce the probability of occurrence. This can be implemented through a continuous quality cycle which may include innovative models of care, more efficient computerized support systems, interventions to improve professional relationships within the staff, measures for patient-centered recovery and training on specific skills that the staff requires.

In particular, an adequate training plan should be developed for health care personnel, for “allied” teams (voluntary associations, social workers, involved police, decision makers). A program for discussing and sharing procedures with all involved actors and agencies must also be implemented.

The actions that cannot be improved in the operational context but are in the hands of the decision makers should be codified and presented to the public. The action plan should also explicitly incorporate structured post-incident reviews, which are recommended in international guidance as a key mechanism for learning, accountability, and continuous improvement following episodes of coercion (51). These reviews allow teams to examine the circumstances of the event, evaluate whether alternative approaches could have been implemented, and identify system-level improvements.

## 5 Analysis of resources

As discussed previously, systemic barriers can be significant elements in the “ecosystem” of involuntary treatment, providing the conditions for such events to occur. Considering that the application of an involuntary health treatment that could have been avoided is a violation of the CRPD, a limited application of the CRPD due to lack of resources means that there may be responsibilities at different decision-making levels.

Thus, in current practice, before the application of an involuntary health treatment, service providers must verify whether all the resources and opportunities that may prevent this measure have been utilized. The CRPD makes clear that the absence of adequate resources does not exempt states or from the obligation to respect legal capacity and avoid involuntary treatment measures; rather, it requires an examination of whether the necessary resources were both available and accessible. In the case where adequate resources were not in place, systematic analysis of which measures could have been provided to avoid the involuntary intervention, and why they were not implemented, should take place.

For example, such resources may include hospital-based rapid response teams capable of delivering immediate clinical stabilization within the inpatient setting, short-stay observation units that provide a time-limited alternative to full admission, enhanced access to specialist consultation–liaison services, and intensive in-hospital follow-up programs designed to maintain engagement among individuals at heightened risk of disengagement from care. In many jurisdictions, the absence or insufficient availability of these services increases the likelihood that situations will escalate to the point where involuntary treatment is considered. Providing concrete alternatives at an early stage is therefore essential to preventing coercion and supporting a rights-based approach to care.

Putting such procedures in place in the first instance will ensure establishment of proper procedures and improvement process and will monitor the resources needed to avoid human rights violations. The action of verification of sentinel events and the whole quality of care cycle focused on these events in the context of human rights is important and outcome studies must focus on these. The verification and study of sentinel events can improve the well-being of people and prevent and mitigate the use of involuntary treatments.

Considering the conceptual nature of this work, the elements presented here should be understood as broader reflections on the potential implications of the proposed model. Viewing involuntary treatment as a preventable sentinel event may strengthen rights-based quality improvement processes, while integrating root cause analysis into existing governance structures can help identify the systemic factors that sustain coercive practices. Meaningful involvement of service users throughout these processes may also support more inclusive and CRPD-aligned forms of shared governance.

At the same time, the model is in an early phase of development and will require piloting to evaluate its feasibility and impact across diverse contexts, where legal and organizational differences may necessitate adaptation. Although not intended as clinical recommendations, these considerations underscore how the proposed approach may guide more structured efforts to monitor and reduce coercive practices. Future collaboration with health systems and policymakers will be essential to assess its real-world implementation.

Moreover, this model was originally envisioned for implementation in the hospital setting; however, with appropriate adjustments, it could also be applied in community contexts where involuntary treatment has been carried out, provided it maintains its reflective orientation, the essential components that constitute it, and its practical impact.

The implementation of such a model will inevitably require adequate resources, including trained staff, time for structured reviews, and the availability of community-based alternatives. In jurisdictions with high rates of involuntary treatment, these resource demands may be substantial and should be carefully considered in any attempt to operationalize the model.

## 6 Service user involvement

As noted earlier in the description of the model, service user participation is intended to be embedded across all stages of the process. This section provides a more detailed examination of how such involvement can be structured and supported in practice. Involvement of and participation by service users is critical for the effective design, implementation, and evaluation of models addressing involuntary treatment as sentinel events. Service user involvement ensures that lived experiences shape the conception and operation of monitoring systems, helping to build rights-compliant, user-centered mental health services (52–55). Meaningful participation goes beyond tokenistic inclusion, empowering users as “experts-by-experience” to influence decisions across all levels of this model, from initial design to ongoing monitoring and evaluation. Engaging service users in the conception, design, and monitoring of sentinel event models offers multiple benefits, including providing unique insights into the root causes of involuntary treatment and alternative approaches, and reducing power imbalances in mental health systems that often influence involuntary treatment. Evidence shows that user participation improves service quality, reduces stigma, and enhances compliance with human rights standards.

From the earliest stages of conceptualizing the sentinel event model, including during the review of the proposed model and the drafting of this article (TZ is a service user researcher), service users and persons with lived experience must play a key role. The input of service users ensures that the model addresses the real-world impacts of involuntary treatment, including trauma, stigma, and systemic barriers, and that mechanisms to explore these factors are part of the design of the model. A participatory design approach could include the formation of a dedicated committee of service users, tasked with co-creating the model alongside mental health professionals, policymakers, and advocates. Such a committee would provide insight into the specific conditions and systemic gaps that lead to coercive practices. Embedding service users in this process will allow the model to better reflect the complex influences and consequences of involuntary treatments. The involvement of service users in the monitoring and analysis of sentinel events is also vital for ensuring accountability and continuous improvement. For example, service user representatives could be included in the teams responsible for root cause analysis of events, as their perspectives can help better identify attitudinal biases, systemic barriers, or overlooked alternatives that may have contributed to the event. Additionally, a model for user-led monitoring could be established, wherein service user organizations are empowered to independently assess the implementation of the sentinel event monitoring system and provide recommendations. Such mechanisms would not only increase transparency, but also build trust and collaboration between users and providers.

To ensure service users can participate meaningfully, it is essential to provide them with adequate training and support. Capacity-building programs should equip users with the knowledge and skills needed to engage in technical discussions, interpret data, and contribute to policy and system-level decision-making. Similarly, training for mental health professionals on the value of user participation can help address stigma and power imbalances that often hinder collaborative efforts. Recent evaluation models underscore how structured and continuous involvement of users and other stakeholders can strengthen the relevance of interventions, align services with real-world needs, and contribute to transformative system-level change (34).

Finally, it is crucial that adequate resources are allocated towards enhancing service user participation in this model: this includes allocating resources specifically for user engagement and fostering environments where their voices are not only heard but acted upon.

## 7 Conclusion

Signing the treaty committed States to change laws in accordance with the principles of the CRPD. However, only a minority of States Parties to the CRPD have enacted legislation aimed at eliminating involuntary treatment, and even fewer have operationalized such reforms. It places healthcare professionals in a situation of ambiguous conflict, if they try to limit or abrogate mandatory healthcare treatments, they risk breaking the law and exposing themselves to being the scapegoat for any negative outcome that may happen to the people to whom the treatment has not been applied mandatorily. If they put in place an involuntary treatment, they may be violating the CRPD that should be itself law of the state. The focus of the debate on involuntary treatment at the individual, clinical level, rather than addressing its root causes, diverts attention from the structural factors and potential areas for systemic intervention. Reframing the long-standing debates surrounding involuntary treatment requires a deliberate shift in discourse toward examining factors that enable involuntary treatment.

What is proposed here is not a reductive approach but to create a feeling of “alert” around events that limit the service user’s freedom, i.e. to define them as “sentinel events” to be analyzed and taken seriously.

Instead, the problem could be addressed by inserting the “sentinel event” initiative as a broader reflection on the operational complexity of translating the dictates of the CRPD into the daily practice of clinical services. Introducing a continuous quality system centered on human rights into clinical practice with the involvement of users would be a solid function on which to base and anchor the continuous training of the health professionals.

## Data Availability

The original contributions presented in the study are included in the article/supplementary material. Further inquiries can be directed to the corresponding author.
